# Comment on: Clinical Validity, Understandability, and Actionability of Online Cardiovascular Disease Risk Calculators: Systematic Review

**DOI:** 10.2196/10093

**Published:** 2018-03-05

**Authors:** Ivan Sisa

**Affiliations:** ^1^ School of Medicine College of Health Sciences Universidad San Francisco de Quito Quito Ecuador

**Keywords:** cardiovascular disease, risk assessment, risk model

I am writing regarding the systematic review about clinical validity, understandability, and actionability of online cardiovascular disease (CVD) risk calculators recently published by Dr Bonner and colleagues [1].

Although Dr Bonner and colleagues used a comprehensive two-step research strategy to identify Web addresses that contained a CVD risk calculator, which led to the identification of 67 Web pages, a very important CVD risk model, the Systematic COronary Risk Evaluation (SCORE) risk assessment model [2], was ignored. Developed by the European Society of Cardiology, this model was derived from 12 European cohort studies (250,000 patients data collected and 3 million person-years of observation) and is based on classical risk factors such as gender, age, total cholesterol, systolic blood pressure, and smoking status. The SCORE risk assessment model should have been included because it satisfies Dr Bonner’s inclusion criteria as it predicts the risk of developing a CVD event and an electronic interactive version of this model is freely available on the European Society of Cardiology's Web page [3].

Furthermore, there are other risk assessment models locally developed in countries such as China, India, and Korea that are not taken into account in this study. Thus, it would have been useful if the authors had added to their research strategy a literature search of review documents focusing on cardiovascular risk assessment as was carried out by Zhao and colleagues [4].

## Abbreviations

CVD: cardiovascular disease
SCORE: Systematic COronary Risk Evaluation

## References

[1] Bonner Carissa, Fajardo Michael Anthony, Hui Samuel, Stubbs Renee, Trevena Lyndal (2018). Clinical Validity, Understandability, and Actionability of Online Cardiovascular Disease Risk Calculators: Systematic Review. J Med Internet Res.

[2] Conroy R M, Pyörälä K, Fitzgerald A P, Sans S, Menotti A, De Backer G, De Bacquer D, Ducimetière P, Jousilahti P, Keil U, Njølstad I, Oganov R G, Thomsen T, Tunstall-Pedoe H, Tverdal A, Wedel H, Whincup P, Wilhelmsen L, Graham I M, SCORE project group (2003). Estimation of ten-year risk of fatal cardiovascular disease in Europe: the SCORE project. Eur Heart J.

[3] European Society of Cardiology.

[4] Zhao D, Liu J, Xie W, Qi Y (2015). Cardiovascular risk assessment: A global perspective. Nat Rev Cardiol.

